# Case report: Mechanical thrombectomy for acute basilar artery occlusion via persistent hypoglossal artery

**DOI:** 10.3389/fneur.2023.1200539

**Published:** 2023-07-27

**Authors:** Xin Zhang, Jiaxiong Wang, Zhipeng Cao, Yingtao Liu, Yi Dong, Xin Cheng, Chao Gao, Yuxiang Gu

**Affiliations:** ^1^Department of Neurosurgery, Huashan Hospital, Shanghai Medical College, Fudan University, Shanghai, China; ^2^National Center for Neurological Disorders, Shanghai, China; ^3^Shanghai Key Laboratory of Brain Function and Restoration and Neural Regeneration, Shanghai, China; ^4^Neurosurgical Institute of Fudan University, Shanghai, China; ^5^Shanghai Clinical Medical Center of Neurosurgery, Shanghai, China; ^6^Department of Neurosurgery, South Yunnan Central Hospital of Yunnan Province, Mengzi, Yunnan, China; ^7^Department of Neurology, Huashan Hospital, Fudan University, Shanghai, China; ^8^Department of Radiology, Huashan Hospital, Fudan University, Shanghai, China

**Keywords:** persistent hypoglossal artery, acute ischemic stroke, basilar artery occlusion, thrombectomy, case report

## Abstract

Persistent hypoglossal artery (PHA) is a rare carotid-vertebrobasilar anastomosis in adults. Here, we report a case of mechanical thrombectomy for acute basilar artery occlusion via the PHA. A 44-year-old man was admitted to our stroke unit with an unstable gait and aphasia for 2 h. The baseline National Institutes of Health Stroke Scale (NIHSS) score was 4, but the clinical symptoms continued to worsen. Computed tomography angiography showed the absence of the basilar artery and an abnormal anastomosis between the anterior and posterior circulation. Clinical symptoms continued to worsen, and endovascular treatment was scheduled. PHA was demonstrated and basilar artery occlusion was confirmed using digital subtraction angiography. Mechanical thrombectomy with a stent retriever and aspiration was performed via the PHA, and modified thrombolysis in cerebral infarction level 3 was achieved. The patient underwent intravenous antiplatelet therapy after the operation, and follow-up neuroimaging revealed multiple small infarcts in the cerebellum and medulla oblongata. The patient was discharged after 10 days for further rehabilitation, with an NIHSS score of 25. At 10 months follow-up, the NIHSS score decreased to 18. Recognition of this rare variation is particularly important for interventional strategy determination and rapid recanalization of basilar artery occlusion.

## Introduction

Basilar artery occlusion (BAO) is the most serious type of acute ischemic stroke. Failure to achieve rapid recanalization can lead to high mortality and disability rates and poor clinical prognosis (1). The safety and efficacy of mechanical thrombectomy (MT) for the treatment of acute large-vessel occlusion in the anterior circulation have been confirmed in many studies (2). Multiple randomized controlled trials (3–6) have shown promising results for MT for acute BAO, with better clinical outcomes and reduced mortality.

Persistent hypoglossal artery (PHA) is a rare carotid-vertebrobasilar anastomosis in adults. Few studies have reported BAO accompanied by PHA. In this case, the patient with BAO received MT, and PHA was confirmed during the operation. Such variation is of great importance in the determination of an endovascular treatment (EVT) strategy for BAO, which includes the establishment of MT access with sufficient supporting strength and the evaluation of compensatory situations.

## Case description

A 44-year-old man was admitted to our stroke unit with unstable gait and dysarthria for 2 h. The baseline National Institutes of Health Stroke Scale (NIHSS) score was 4 and the Modified Rankin Scale (mRS) score was 1. As the clinical symptoms progressively worsened, the NIHSS score increased to 33 and the mRS score to 4 within 95 min. The patient had a heavy smoking history and primary hypertension treated with nifedipine. In addition, the patient had a history of undefined intracranial hemorrhage; therefore, thrombolytic therapy was a contraindication.

**Figure 1:**
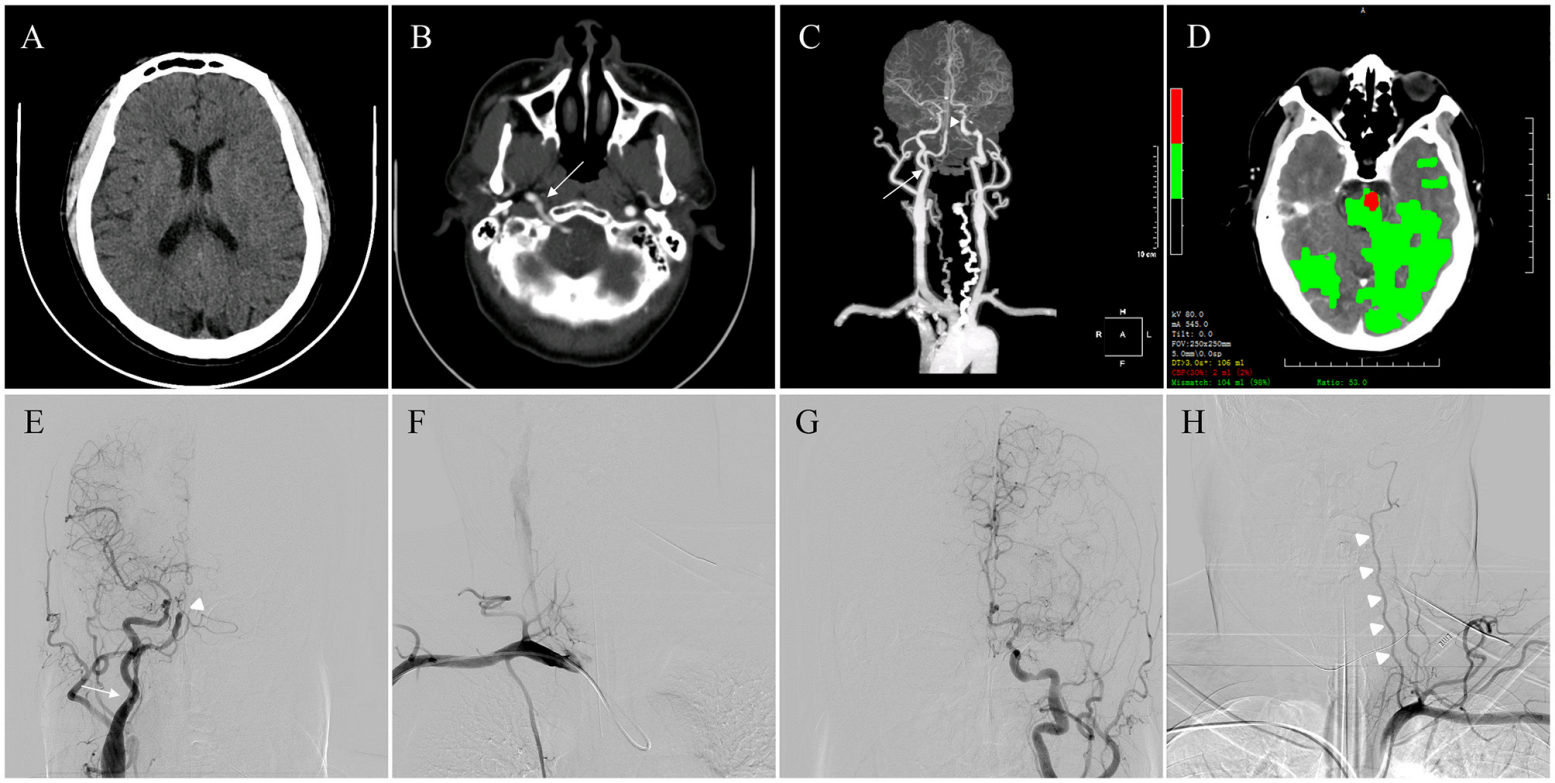
Preoperative multimodal neuroimaging evaluation. **(A)** Intracranial hemorrhage was excluded through computed tomography (CT). **(B)** Cross-sectional contrast CT revealed abnormal anastomosis (arrow) between the internal carotid artery (ICA) and the intracranial segment of the vertical artery (VA) located in the atlantooccipital space. **(C)** CT angiography (anterior-posterior view) showed basilar artery occlusion (arrowhead) and that right VA (arrow) originated from the ICA. **(D)** Volumes of infarct core (red area) and penumbra (green area) were evaluated automatically from CT perfusion to calculate the mismatch volume. **(E)** Right hemispheric angiography showed complete occlusion of the middle segment of the basilar artery (arrowhead) and that persistent hypoglossal artery (PHA) (arrow) originated from the right ICA. **(F)** Right subclavian artery angiography showed the absence of VA origin. **(G)** Left middle cerebral artery occlusion was observed with collateral vessel formation. **(H)** Slender left VA (arrowheads) demonstrated in left hemispheric angiography.

Multimodal computed tomography (CT) was performed, and intracranial hemorrhage was excluded (Figure 1A). CT angiography (CTA) showed the absence of the basilar artery (BA) and an abnormal anastomosis between the internal carotid artery (ICA) and the intracranial segment of the vertebral artery (VA) located in the hypoglossal canal (Figures 1B, C). The size of the area with cerebral blood flow >30% and delay time < 3 s were determined using CT perfusion. The mismatch volume was 104 mL (Figure 1D), determined using MIStar software (Apollo Medical Imaging Technology, Melbourne, Australia). Digital subtraction angiography was performed via the right radial access and showed complete BAO with a modified Thrombolysis in Cerebral Infarction (mTICI) level of 0. PHA originated from the right ICA and anastomosed to the right VA. Right subclavian artery angiography revealed hypoplasia of VA origin. Left middle cerebral artery occlusion with collateral vessel formation and a slender left VA were also observed (Figures 1E–H).

## Treatment

**Figure 2:**
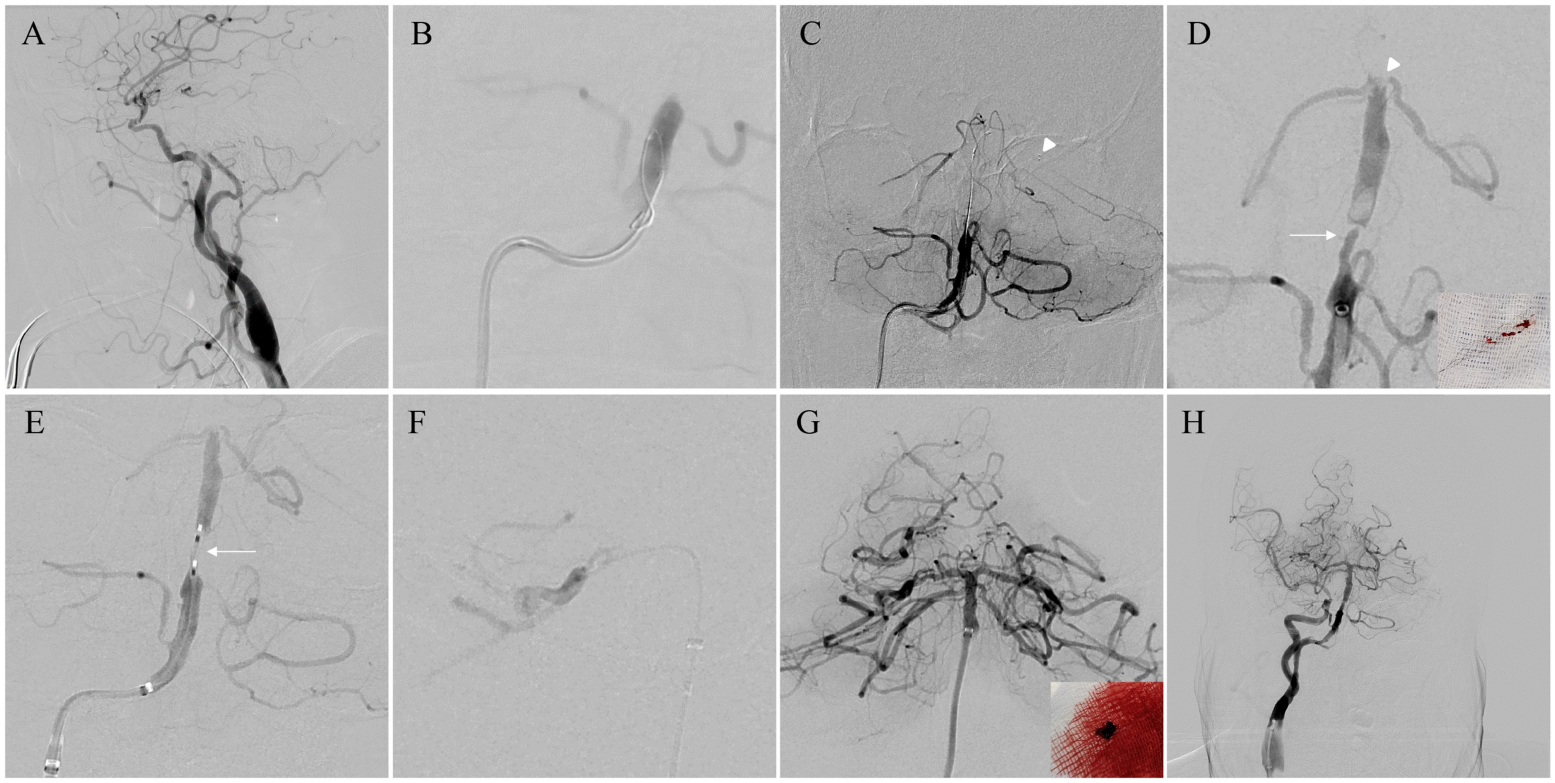
Surgical process. **(A)** Lateral view as a working projection for the establishment of access for thrombectomy. **(B)** Angiography via the microcatheter to confirm the real lumen of the distal basilar artery (BA). **(C)** The distal part of the stent retriever (arrowhead) was deployed from the left posterior cerebral artery. **(D)** After the first thrombectomy, part of the clot was removed with the stent (right corner), and cerebral angiography showed the migration of residual thrombosis to the tip of the BA (arrowhead) and severe stenosis in the BA trunk (arrow). **(E)** Angioplasty with a 3 × 9 mm balloon (arrow) in the stenotic portion of the BA. **(F)** The second thrombectomy was performed from the right persistent hypoglossal artery (PCA) with proximal aspiration. **(G)** Complete recanalization of the BA and the clot from the aspiration catheter (right corner). **(H)** Modified Thrombolysis in Cerebral Infarction (mTICI) level 3 forward flow was confirmed after 20 min.

Great difficulties were encountered in the establishment of MT access with sufficient supporting strength via transradial access; therefore, transfemoral access was selected. A triaxial system, including a five French aspiration catheter (Navien, Medtronic, Irvine, CA, USA), microcatheter (Prowler Select Plus, Cerenovus, Bridgewater, NJ, USA), and stent retriever (Solitaire AB, Medtronic), was chosen for MT. A 4 × 20 mm Solitaire AB stent was deployed from the P1 segment of the left posterior cerebral artery (PCA) via the PHA for thrombectomy. Angiography showed thrombotic migration to the tip of the BA and severe stenosis in the BA trunk. After angioplasty with a 3 × 9 mm noncompliant balloon (Gateway, Stryker Neurovascular, Kalamazoo, Michigan), thrombectomy was performed again from the right PCA with proximal aspiration via a 5 French distal access catheter (Silver Snake, TonBridge Medical, Zhuhai, China). Complete recanalization of BA flow was achieved at an mTICI level of 3. Postoperative CT revealed no obvious intracranial hemorrhage, and a loading dose of tirofiban was administered (Figures 2A–H).

## Outcome and follow-up

**Figure 3:**
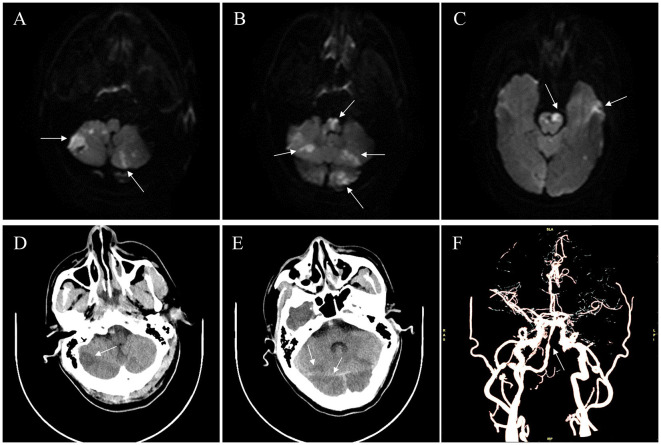
Neuroimaging follow-up. **(A–E)** Postoperative follow-up revealed multiple small newly developed infarcts (arrows) in the cerebellum and medulla oblongata on diffusion weighted imaging and computed tomography (CT). **(F)** Postoperative CT angiography confirmed the general patency of the BA trunk, with slight stenosis (arrow).

The patient received half loading-dose of intravenous antiplatelet therapy (Tirofiban) and the systolic blood pressure was strictly maintained between 130 and 140 mmHg after the operation. Postoperative neuroimaging follow-up revealed multiple small, newly developed infarcts in the cerebellum and medulla oblongata (Figures 3A–E). CTA performed 3 days after the operation showed good patency of the BA trunk (Figure 3F). The patient was discharged after 10 days for further rehabilitation, with an NIHSS score of 25 and a mRS score of 5. At 10 months follow-up, the NIHSS score decreased to 18 and the mRS score decreased to 4.

## Discussion

Few studies have reported the occurrence of BAO accompanied by PHA (Table 1). During early development of the human embryo, various anastomotic channels exist between the carotid artery and vertebrobasilar system, which play critical roles in irrigating the posterior circulatory bed before it fully develops (7). PHA is usually involved in the 12 to 14 mm embryonic stage. Rarely, it fails to regress and persists into adult life, with an incidence of 0.03–0.09% (8). PHA is generally accompanied by other anomalies such as hypoplasia of the VA or PCA. Due to its unique anatomical and hemodynamic characteristics, PHA is a potential risk factor of posterior circulation ischemic events (9). However, cases of PHA combined with acute BAO have rarely been reported.

**Table 1:** Previous cases of BAO accompanied by PHA.

**Previous literature**	**Age (years)/sex**	**Clinical presentation**	**Medical history**	**Baseline NIHSS**	**Imaging findings**	**TOAST type**	**EVT strategy**	**Outcome/ follow up**
Kawano et al. (11)	76/Male	Consciousness lost	Severe dilated cardiomyopathy and cardiac dysfunction	N/A	PHA, BAO and occlusion of left ICA and VA	Cardioembolism	N/A	Died after 3 days
Voronovich et al. (12)	48/Male	Unresponsive with flaccid extremities	Atrial septal defect repairment	28	PHA and BAO	Cardioembolism	Aspiration	Aphasia at discharge
See et al. (8)	70/Female	Nausea, dizziness and dysphasia on awakening, followed by left hemiparesis and decreased consciousness.	Ischemic cardiomyopathy and cardiac defibrillator implantation	From 4 to 22	PHA, BAO and atherosclerotic changes at ICA	Stroke of undetermined cause	Aspiration	Mechanical ventilation and appropriate motor function improvement of limbs at discharge
Park et al. (13)	83/Female	Acute unresponsive mental deterioration onset	Hypertension	20	PHA and BAO	Stroke of other determined cause	Aspiration	Drowsy and mild dysarthria after 1 month
Present study	44/Male	Unstable gait and dysarthria	Smoking, hypertension and undefined intracranial hemorrhage	From 4 to 33	PHA and BAO	Large-artery atherosclerosis	Stent retriever, aspiration and angioplasty	NIHSS score was 25 at discharge and decrease to 18 at 10 months follow-up

BAO, basilar artery occlusion; PHA, persistent hypoglossal artery; NIHSS, National Institutes of Health Stroke Scale; TOAST, Trial of Org 101072 in Acute Stroke Treatment; EVT, endovascular treatment; ICA, internal carotid artery; VA, vertebral artery.

Earlier recanalization has been correlated with a better prognosis for acute BAO. Owing to its various clinical symptoms, advanced CT evaluations are essential to select suitable cases for patients with BAO (10). Based on the MIStar software, the ischemic penumbra and core infarction could be identified, even though the diagnostic value may be limited because of the influence of the blood flow velocity and the bony structure of the posterior fossa. In addition, CTA is helpful in distinguishing potential variations so that appropriate access catheters can be selected effectively to shorten recanalization time. Hence, the application of advanced CT evaluations is particularly important in surgical decision-making for BAO combined with PHA.

Previous studies (11–13) on BAO accompanied by PHA have mostly attributed it to cardioembolic stroke, and aspiration alone has a high rate of recanalization (Table 1). In this case, the mechanism of BAO was considered to be ischemic stroke based on intracranial atherosclerotic stenosis (ICAS), which is highly prevalent in East Asian populations. Although bridging intravenous treatment may confer benefits for ICAS, the patient had a history of intracranial hemorrhage; therefore, direct MT was selected. To the best of our knowledge, this is the first report of the treatment of BAO via PHA using a stent retriever with aspiration. Similar to the pathogenesis of ICAS, rescue angioplasty after MT is essential for maintaining forward blood flow. More importantly, although the BA was completely recanalized with an mTICI level of 3, embolic debris migration and limited collateral circulation may have resulted in a very poor prognosis. Despite the aforementioned methods, flow control in the proximal part of the ICA with a balloon-guiding catheter may also be more effective and should be seriously considered in subsequent clinical practice (14).

## Conclusion

BAO accompanied by PHA is extremely rare, and relevant literature is lacking. Preoperative multimodal CT evaluations are helpful in identifying such vascular variations to determine an interventional strategy for earlier recanalization. Proximal flow control, aspiration with a balloon-guiding catheter, and the use of intra-arterial alteplase or tirofiban may improve the clinical outcomes of ischemic stroke based on ICAS.

## Data availability statement

The raw data supporting the conclusions of this article will be made available by the authors, without undue reservation.

## Ethics statement

The studies involving human participants were reviewed and approved by the Institutional Review Board (IRB) of Huashan Hospital, Fudan University, China (approval number KY2015-256). The patients/participants provided their written informed consent to participate in this study. Written informed consent was obtained from the individual(s) and/or minor(s)' legal guardian/next of kin for the publication of any potentially identifiable images or data included in this article.

## Author contributions

XZ, JW, ZC, YL, YD, XC, CG, and YG contributed to the conception and design, acquisition and interpretation of the case data, drafting and revision of the article, particularly for important intellectual content, final approval of the published version, and agreement to be accountable for the accuracy or integrity of the article. All authors contributed to the manuscript and approved the submitted version.
